# PRDX2 in Myocyte Hypertrophy and Survival is Mediated by TLR4 in Acute Infarcted Myocardium

**DOI:** 10.1038/s41598-017-06718-7

**Published:** 2017-08-01

**Authors:** Xian Jin, Chengjun Chen, Dandan Li, Qian Su, Yanwen Hang, Peng Zhang, Wei Hu

**Affiliations:** Department of Cardiology, Shanghai Minhang Hospital, Fudan University, Shanghai, People’s Republic of China

## Abstract

Peroxiredoxin 2 (PRDX2) is an antioxidant and molecular chaperone that can be secreted from tumor cells. But the role of PRDX2 in acute myocardial infarction (AMI) is not clear. In the current study, we demonstrate the role of PRDX2 from clinical trials, H9c2 cells and in a mouse model. ELISA analysis shows that serum concentrations of VEGF and inflammatory factor IL-1β, TNF-α and IL-6 were increased in AMI patients compared to a control group. The expression of PRDX2 was also upregulated. *In vivo* experiments show that the expression of PRDX2 inhibits hypoxia-induced oxidative stress injury to H9c2 cells. However, PRDX2 expression promotes TLR4 mediated inflammatory factor expression and VEGF expression under hypoxia conditions. PRDX2 overexpression in H9c2 cells also promotes human endothelial cell migration, vasculogenic mimicry formation and myocardial hypertrophy related protein expression. The overexpression of PRDX2 inhibits ROS level and myocardial injury after AMI but promotes inflammatory responses *in vivo*. Immunocytochemistry and immunofluorescence analysis show that overexpression of PRDX2 promotes angiogenesis and myocardial hypertrophy. Taken together, our results indicate that PRDX2 plays two roles in acute infarction – the promotion of cell survival and inflammatory myocardial hypertrophy.

## Introduction

Peroxiredoxins (PRDXs) are a ubiquitous family of antioxidant enzymes that act as important regulators of redox signaling but have also been implicated in several diseases including cancers, neurodegenerative diseases and inflammatory diseases^1, 2^. They are, therefore, often viewed as both therapeutic tools and therapeutic targets^3–6^. In mammals, there are currently six known PRDX isozymes, PRDX1–6, that contain either one or two redox-active cysteine residues that are sensitive to oxidation by H_2_0_2_^7^. PRDX2 is a 25 kDa protein that is abundant in the cytosol of a wide range of tissues and the second most abundant protein to hemoglobin in erythrocytes. It is thought to have an important function in protecting blood cells from oxidative stress. PRDX2 knockout mice exhibit hemolytic anemia^8^ and overexpression of PRDX2 is known to protect leukemia cells from apoptosis^9^. Furthermore, overexpression of PRDX2 protects cardiomyocytes from oxidative stress-induced cell death and apoptosis, and its downregulation counteracts this protective effect, indicating it may function in cardiac protection against oxidative stress^10^.

PRDXs can induce the expression of inflammatory cytokines through the activation of Toll-like receptors (TLRs) and are known to be key initiators of post-ischemic inflammation^11–13^. TLR signaling is thought to mediate cytokine release in cardiomyocytes following hypoxia-induced proinflammatory gene expression^14^. The TRL4-mediated release of cytokines in cardiomyocytes results in inflammation-induced cardiac injury through regulating the expression of TNF-α, IL-6, and chemotactic cytokines ligands^15^. In addition, the TLR4-mediated pathway in combination with PI3K/Akt/mTOR signaling were found to be involved in the development of cardiac hypertrophy *in vivo* ^16^.

Vascular endothelial growth factors (VEGF) are a subfamily of signal proteins that stimulates vasculogenesis and angiogenesis^17^. They are also involved in the restoration of oxygen to tissues in hypoxic conditions^18^. There are five different isoforms of VEGF, VEGFA–D and placenta growth factor, that interact with three receptors, VEGFR1-3^19^. When PRDX2 is downregulated, VEGFR2, which has an oxidation-sensitive cysteine residue whose reduced state is regulated by PRDX2, is unable to stimulate VEGF^20, 21^. PRDX2 is thought to interact with VEGFC which can promote myocyte hypertrophy and survival in the infarcted myocardium^22^.

In this study, we investigate the diverse influence of PRDX2 on TLR4 and VEGF in myocyte hypertrophy and acute myocardial infarction (AMI) from data acquired through clinical trials, H9c2 cells and in a mouse model system.

## Materials and Methods

### Ethics Statement

All animal/human experiments were performed with the approval of the animal/human ethics committee of the China of Shanghai Minhang District Central Hospital. Experiments were conducted by following established guidelines for animal/human care and were approved by the animal/human ethics committee of the China of Minhang District Central Hospital.

### Animals

Wild-type (WT) and TLR4^lps-del^ (TLR4^−/−^) C57BL/10 mice were purchased from the Model Animal Research Center of Nanjing University (MARC). Six-week-old WT and TLR4^−/−^ mice were used in this study. All animals were treated in accordance with the Guide for the Care and Use of Laboratory Animals.

### Reagents

Antibodies: CD31, PRDX2, GAPDH, NFκB, Bax, caspase-3, and Bcl-2 were from Sigma (St. Louis, MO, USA). Dulbecco’s modified Eagle’s medium (DMEM, high glucose) serum-free endothelial basal medium (EBM) and fetal bovine serum were from Nego (Shanghai, China). Cell lysis buffer (10×) was obtained from Cell Signaling Technology (Danvers, MA, USA). The RT-PCR kit was purchased from TOYOBO (Shanghai, China). Other reagents include DAPI (Roche, Germany), hematoxylin and eosin (H&E, Toronto Chemicals, Toronto, Canada), and trypsin (Sigma). All pairs of real-time PCR primers were synthesized by Shenggong Biotechnology (Shanghai, China). Other chemicals and reagents were of analytical grade.

### Clinical specimen collection and ethical statement

Serum samples from 77 AMI patients or 30 healthy volunteers were obtained at the Shanghai Central Hospital of Minhang District between July 2015 and October 2016. The AMI cases were identified from electronic medical records. Of the 77 patients, 69 were male and eight were female. Sixteen patients were diagnosed with a history of diabetes. Forty-two patients were diagnosed with hypertension. Thirty-three and 10 patients were defined as smokers or drinkers, respectively. All the samples were frozen in liquid nitrogen immediately. This study was approved by the Ethics Committee of Shanghai Central Hospital of Minhang District and written informed consent was obtained from all patients.

### Cell culture and establishment of hypoxic culture conditions

Embryonic rat heart-derived H9c2 cells and human umbilical vein endothelial cells (HUVEC) were purchased from the Cell Bank of the Chinese Academy of Sciences (Shanghai, China). H9c2 cardiomyocytes were cultured in high-glucose DMEM supplemented with 10% (v/v) fetal bovine serum, 50 U/ml penicillin, 50 μg/ml streptomycin, and 2 mM L-glutamine. Cells were maintained at 37 °C in an atmosphere of 95% air and 5% CO_2_. After reaching 80–90% confluence, H9c2 cells with or without PRDX2 overexpression were cultured with serum-free medium in a hypoxia chamber (Thermo, Dreieich, Germany) for the indicated time periods. The hypoxic atmosphere was maintained at 37 °C with 2% O_2_/93% N_2_/5% CO_2_. Cells were then removed from the hypoxic chamber and used for further experiments. For other experiments, H9c2 cells exposed to a hypoxic atmosphere for 6 h were used.

### Cell viability assay

The H9c2 cells were seeded in 96-well plates at a concentration of 1 × 10^4^ cells/well. After exposure in a hypoxic atmosphere for the indicated time periods, cell viability was detected by using a CCK-8 kit. The absorbance of each well was determined by a microplate reader at 450 nm.

### Determination of ROS levels

ROS production in H9c2 or myocardial tissue was measured using 2′,7′-dichlorofluorescein diacetate (DCF-DA, Molecular Probes, Eugene, OR, USA). After hypoxia treatment, cells or myocardial tissue from the infarction area were washed in PBS and co-cultured with 10 μM DCFH-DA at 37 °C in an incubator for 20 min with gentle shaking in the dark. The images were obtained with a fluorescence microscope (Nikon) at 488 nm excitation and 590 nm emission. The mean fluorescence intensity (MFI) were measured with ImageQuant version 5.2 (Molecular Dynamics).

### Flow cytometry

Cells were collected after hypoxia treatment and washed with PBS, and then 1 × 10^5^–5 × 10^5^ cells were suspended in 500 μl binding buffer. FITC-annexin V (5 μl) and 5 μl propidium iodide (PI) were added to the cell suspension. The cells were incubated at room temperature for 10 min in the dark. The samples were analyzed by flow cytometry (Becton-Dickinson).

### Western blotting

Total proteins from cells or myocardial tissue were extracted and quantified. Then, 30–50 μg of each sample was separated using 10% SDS-PAGE gel electrophoresis (stacking gel 50 V, separating gel 100 V), and transferred to a nitrocellulose membrane (100 V, 75 min). Membranes were then blocked and incubated with primary antibodies followed by secondary antibodies. HRP-labeled secondary antibodies were detected using chemiluminescence, and the grayscale of the protein bands was analyzed using Gel-pro Image Analysis Software (Media cybernetics, Rockville, MD, USA).

### Construction and transfection of the PRDX2 vector and siRNAs

PRDX2 full-length cDNA from mice or rat were generated by PCR using forward primer: 5′-GGCAGATCTATGGCCTCCGGCAACGCGCA-3′, and reverse primer: 5′-CAGGAATTCTCAGTTGTGTTTGGAGAAGT-3′. The amplified fragment was then subcloned into a pZsGreen1 plasmid (GeneChem, Shanghai, China) after *Bgl*II/*EcoR*I restriction enzyme digestion. Transfection of H9c2 cells was carried out using AAV9 (Adeno-associated virus 9, Invitrogen, Carlsbad, CA, USA). For knockdown of PRDX2 and TLR4, siRNA molecules (siPRDX2 and siTLR4) were synthesized by GenScript Co., Ltd. (Nanjing, China). Their target sequences were 5′-AGGGGCCTCTTTATCATCGATGC-3′ (siPRDX2) and 5′-GGACCTATGAGACCTTCAATT-3′ (siTLR4). The scramble sequence were: 5′-AGCCCGCATCCGATGC-3′ (SCR). H9c2 cells were dispersed into collagen I-coated six-well plates at 5 × 10^4^ cells/well and cultured overnight under normal conditions. The following day, cells were transfected with siRNA or in serum-free medium using oligofectamine reagent (Invitrogen) for 4 h at 37 °C with 5% CO_2_. After 4 h transfection, an equal volume of medium, supplemented with 20% FBS, was added and the transfectants were cultured until further analyses.

### Real-time PCR analysis

RNA was isolated from H9c2 cells or myocardial tissue using TRIZOL (Invitrogen). cDNA was synthesized from 1 μg of total RNA, using oligo dT18 primers and superscript reverse transcriptase in a final volume of 21 μl. For standard PCR, 1 μl of the first strand cDNA product was used as a template for PCR amplification with Taq DNA polymerase (TaKaRa). PCR amplification proceeded as follows: 30 thermocycles of 94 °C for 30 s, 55 °C for 30 s, and 72 °C for 30 s, using oligonucleotides specific for PRDX2 (sense, 5′-CACGGCCACCGCCGTGGTGG-3′; anti-sense, 5′-TCCGTGGGGTATTGATCCAG-3′), NFκB (sense, 5′-GCCTTATGTGGAGATCATCG-3′; anti-sense, 5′-CACAAGTTCATGTGGATGAG-3′), VEGF (sense, 5′-CCCAGGCTGCACCCACGACA-3′; anti-sense, 5′-CGCACTCCAGGGCTTCATCG-3′), ANP (sense, 5′-GGCCATATTGGAGCAAATCC-3′; anti-sense, 5′-CTATCGGAGGGGTCCCAGGG-3′) and GAPDH (sense, 5′-CACAGTCAAGGCCGAGAATG-3′; anti-sense, 5′-TCGTGGTTCACACCCATCAC-3′).

### Transwell migration assays

The transwell migration assay was performed using transwell membrane (8 μm pore size, 6.5 mm diameter) from Corning Costar (New York, NY, USA). The bottom chambers of the Transwell were filled with migration-inducing medium from cultured H9c2 cells which were exposed to hypoxic conditions for 6 h then cultured in normal conditions for another 24 h. The top chambers were seeded with 10^5^ HUVEC cells. After 16 h, cells migrated through pores to the bottom surface of the transwell and were fixed with 10% formaldehyde, stained with 0.5% crystal violet, and counted under an Olympus inverted microscope. Ten randomly selected microscopic fields were counted for each group. The invasion assays were performed using similar transwell membranes coated with Matrigel (Chemicon International, Temecula, CA, USA). Data were analyzed using ImageJ 1.42q software (National Institute of Health).

### Evaluation of PRDX2 overexpression in H9c2 cells on HUVEC angiogenesis in hypoxic conditions

To assess the effect of H9c2 cells on HUVEC tube formation in hypoxia conditions. H9c2 cells overexpressing PRDX2 and normal H9c2 cells were exposed to hypoxic conditions for 6 h (a control group was exposed to normal conditions) then cultured in normal conditions for another 24 h, then the media were harvested and 2 × 10^4^ HUVECs were added and seeded on Matrigel (~50 μl Matrigel was seeded into the cold wells of a 96-multiwell plate maintained at 4 °C). Matrigel jellification was performed at 37 °C for 30 min. On the 2nd day after seeding, the number of tube formations were counted at 10× magnification by inverted microscopy.

### Myocardial Infarction Model

Myocardial Infarction was produced by surgical ligation of the left anterior descending coronary artery (LAD). After anesthetized by ketamine hydrochloride (50 mg/kg) and diazepam (5 mg/kg), the chest was opened at the left fourth inter costal space and LAD was ligated by a 6-0 silk suture 1 mm below the tip of the left atrial appendage. Successful ligation was verified by color change and electro-chemical grinding. In order to analyse the effect of PRDX2 on myocardial tissue, 100 μl AAV9-pZsGreen1 was administered through left-ventricle cavity injection with a titer of 1 × 10^8^ TU/ml.

### Measurement of Myocardial Infarct Size

Myocardial infarct size was evaluated by 2,3,5-triphenyl-2H-tetrazolium chloride (TTC) staining. Hearts were reperfused with PBS injected into the aorta after being ligated for 2 months. The heart was quickly excised, frozen in liquid nitrogen before being cut transversally into 1 mm thick slices, and incubated in 1% TTC (Sigma, USA) at 37 °C for 10 min. The area stained red by TTC represents ischemic but viable tissue. Infarcted myocardium was not stained by TTC and is more pale than the TTC stained area. The area of infarct size (IS) and area at risk (AAR) were measured digitally using Image Pro Plus software (Media Cybernetics). IS and AAR were expressed as percentages of the left ventricular area (IS/LV and AAR/LV, respectively).

### Determination of Myocardial Apoptosis

Myocardial apoptosis was determined by terminal deoxynucleotidyl transferase-mediated dUTP-biotin nick end labeling (TUNEL) staining using a commercially available kit (In SituCell Death Detection Kit; Roche Diagnostics) for apoptotic cell nuclei. The apoptotic index (AI) was determined as the number of TUNEL-positive nuclei divided by the total number of nuclei stained with DAPI from a total of 40 fields per heart (n = 5). Heart tissue samples for the determination of apoptosis activity-related proteins were obtained from the margin of infarct (peri-infarct) areas. Myocardial caspase-3, Bax, and Bcl-2 levels were examined by western blots.

### ELISA

Tumor necrosis factor-alpha (TNF-a), interleukin (IL)-1β, IL-6, PRDX2 and VEGF secretion in cell culture supernatants or serum were measured by ELISA, according to the manufacturer’s standard protocols (eBioscience, San Diego, CA). Absorbance was read on a Multiscan FC plate reader and analyzed with Skan It for Multi scan FC software (Thermo Scientific, Schwerte, Germany).

### Hematoxylin and Eosin staining

Cardiomyocytes or myocardial tissue were fixed and stained with H&E; cell surface area was calculated by measuring 50 random cells with Image Pro Media Cybernetics.

### Statistical analysis

All data are reported as the mean ± SD. Statistical analysis was performed using the Student’s two-tailed unpaired *t*-test for comparisons between two groups. In all cases, p < 0.05 was considered to be statistically significant.

## Results

### Clinical characteristics of patients with acute myocardial infarction

Table 1 summarizes the demographic characteristics and serum cytokine levels of the 77 AMI patients and 30 healthy subjects in the study. Compared to healthy subjects, the AMI patients have significantly higher levels of VEGF (*p* = 0.003) and PRDX2 (*p* = 0.043). AMI patients also have significantly higher levels of CD34^+^ (*p* = 0.005) and higher serum levels of the cytokines interleukin IL-1β (*p* = 0.012), TNF-α (*p* = 0.002), and IL-6 (*p* = 0.005). Blood glucose, brain natriuretic peptide, and serum triglyceride levels were also significantly elevated in AMI patients (*p* = 0.005, 0.031, and 0.007, respectively). There was no significant difference found in serum total cholesterol, HDL cholesterol and LDL cholesterol levels (*p* = 0.105, 0.533, 0.208, respectively).

**Table 1:** Demographic characteristics and serum cytokine levels of AMI cases and controls.

Characteristics	AMI cases (n = 77)	Control (n = 30)	*p*-value
	N (%) or Mean ± SD		
Age (year)	62.71 ± 14.69	60.87 ± 9.97	0.02
Gender			
Men	69 (89.6%)	23 (76.7%)	0.083
Women	8 (10.4%)	7 (23.3%)	0.083
Diabetes	16 (20.8%)	5 (16.7%)	0.63
Current smoker	33 (42.9%)	16 (53.3%)	0.329
Current drinker	10 (13.0%)	7 (23.3%)	0.188
Hypertension	42 (54.5%)	12 (40.0%)	0.176
TC (mg/dl)	4.29 ± 1.03	4.01 ± 0.84	0.105
BG on admission (mg/dl)	6.32 ± 2.32	5.32 ± 1.29	0.005
BNP (pg/ml)	2217.71 ± 5433.07	603.1 ± 2058.38	0.031
Serum triglycerides (mmol/L)	1.57 ± 1.08	1.34 ± 0.49	0.007
Serum HDL cholesterol (mmol/L)	0.98 ± 0.25	1.12 ± 0.32	0.533
Serum LDL cholesterol (mmol/L)	3.09 ± 0.98	2.63 ± 0.81	0.208
VEGF (pg/ml)	286.2856.34 ± 43.35	223.33 ± 32.45	0.003
PRDX2 (pg/ml)	78.35 ± 8.26	56.34 ± 6.29	0.043
CD34^+^ (Number/μl)	2.52 ± 0.33	1.32 ± 0.42	0.005
IL-1β (pg/ml)	8.14 ± 1.24	4.32 ± 0.86	0.012
TNF-α (pg/ml)	24.56 ± 2.34	15.48 ± 1.87	0.002
IL-6 (pg/ml)	48.23 ± 5.36	26.24 ± 3.58	0.005

Values are mean ± SD, p < 0.05 means that the difference between two groups are significant. AMI, acute myocardial infarction; TC, total cholesterol; HDL-C, high-density lipoprotein cholesterol; BG, blood glucose; BNP, brain natriuretic peptide; PRDX2, peroxiredoxin 2; VEGF, Vascular Endothelial Growth Factor; IL-1β, Interleukin-1β; TNF-α, tumor necrosis factor-α; IL-6, Interleukin-6.

### Effects of hypoxia-induced oxidative stress on H9c2 cells

**Figure 1:**
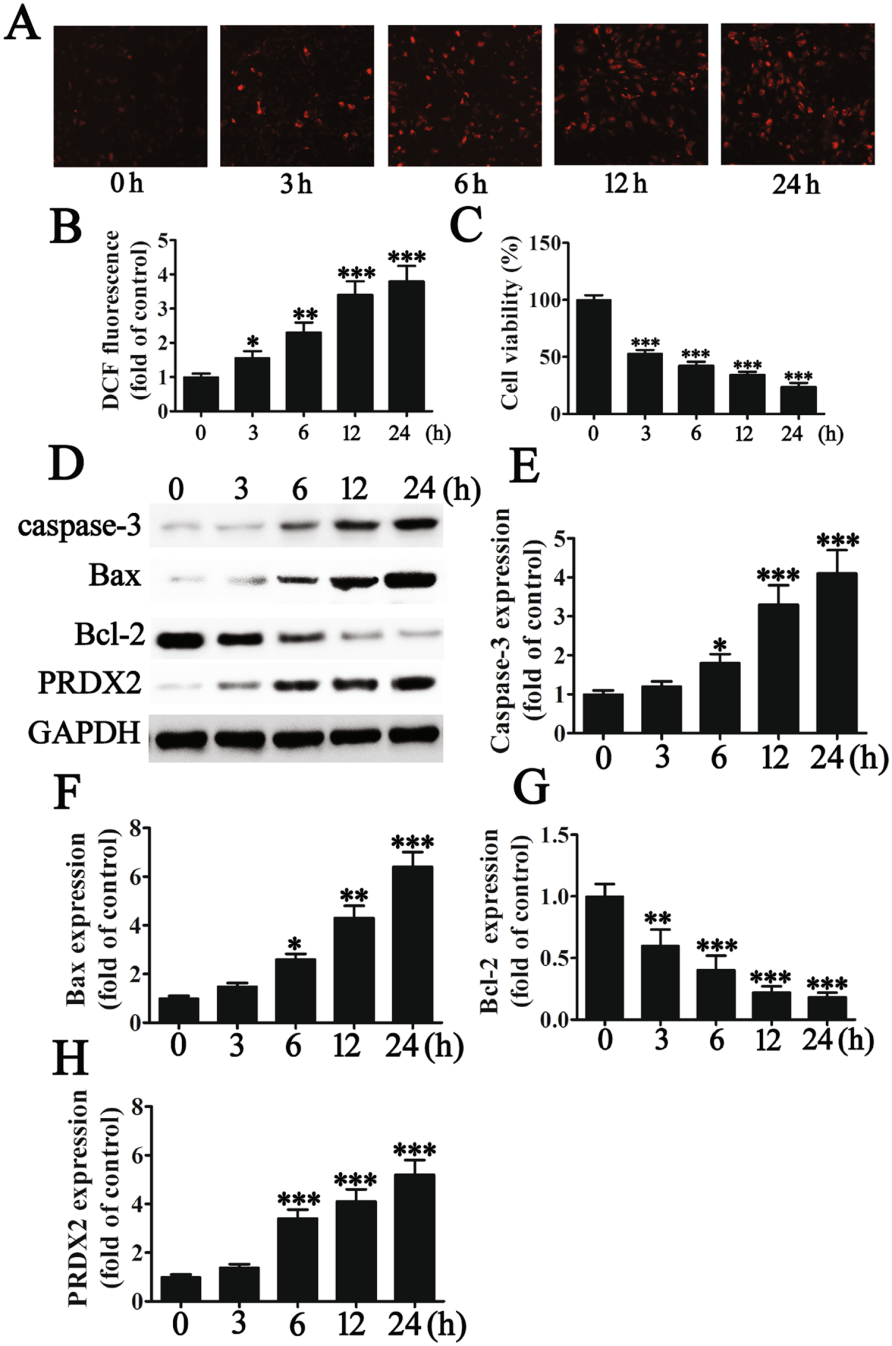
Hypoxia-induced oxidative stress inhibits H9c2 viability and promotes H9c2 apoptosis. H9c2 were exposed to hypoxia conditions for 0, 3, 6, 12 or 24 h before cell activity, ROS production and apoptosis-related protein were measured. (**A** and **B**) Mitochondrial ROS production in H9c2 cells at different times of hypoxic treatment. The data are presented as means ± SD of the mean from three separate cell experiments. ***p < 0.001 vs control. Magnification 200×. (**C**) The effects of hypoxia treatment on H9c2 cell viability. The data are presented as means ± SD of the mean from three separate cell experiments. *p < 0.05, **p < 0.01, ***p < 0.001 vs control. (**D**–**H**) The expression of apoptosis-related proteins was measured by western blot. GAPDH was used as a loading control. The results show that hypoxia treatment promotes the expression of caspase-3 (**E**) and Bax (**F**) in H9c2 cells but inhibits Bcl-2 expression (**G**). The results also show that hypoxia promotes the expression of PRDX2 which may be related to anti-oxidative stress. The data are presented as means ± SD of the mean from three separate cell experiments. *p < 0.05, **p < 0.01, ***p < 0.001 vs control.

We exposed H9c2 cells to hypoxia conditions for up to 24 h and then measured cell activity, ROS production and apoptosis-related proteins (Fig. 1). Mitochondrial ROS production in H9c2 cells increased significantly under hypoxic conditions after 3 h (*p* < 0.05) when measured by DCF fluorescence and continued to increase for 24 h (*p* < 0.001) (Fig. 1A,B). Hypoxia resulted in reduced H9c2 cell viability (Fig. 1C) and an apoptotic-related protein profile after 24 h with significantly promoted expression levels of caspase-3 and Bax (all *p* < 0.001at 24 h vs control) while levels of Bcl-2 were inhibited (*p* < 0.001 vs control) (Fig. 1D–G). Hypoxia also promotes the expression of PRDX2 (p < 0.001), which is thought to be related to anti-oxidative stress (Fig. 1H). Overall these results demonstrate that hypoxia-induced oxidative stress inhibits H9c2 viability and promotes H9c2 apoptosis.

### PRDX2 expression inhibits hypoxia-mediated oxidative stress injury to H9c2 cells

**Figure 2:**
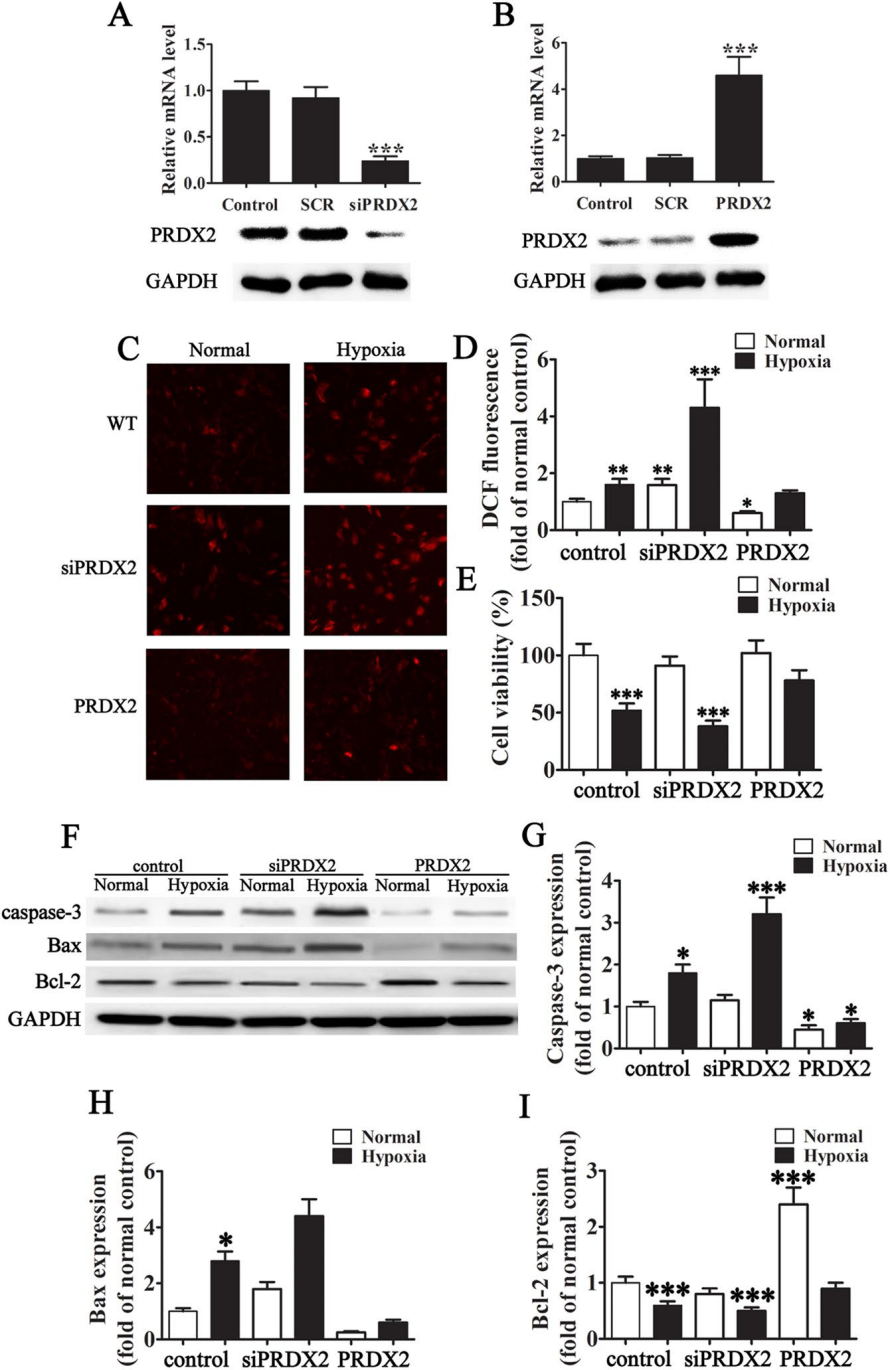
The expression of PRDX2 inhibit hypoxia-mediated oxidative stress injury to H9c2. (**A**) H9c2 cells were transfected with PRDX2 siRNA (siPRDX2) and control siRNA (siSCR) at a concentration of 80 pmols. Cells were harvested 36 h after the transfection, and the silencing effect on PRDX2 mRNA levels was determined by RT-PCR. The silencing effect of PRDX2 siRNA at the protein level was determined 48 h post-transfection using western blotting. The data are presented as means ± SD of the mean from five separate cell experiments. ***p < 0.001 vs control. (**B**) H9c2 cells were transfected with a PRDX2 overexpression vector and control (SCR). Cells were harvested 48 h after the transfection, and the PRDX2 mRNA levels were determined by RT-PCR. The overexpression effect of PRDX2 at the protein level was determined 48-h post-transfection using western blotting. GAPDH was used as a loading control. The data are presented as means ± SD of the mean from five separate cell experiments. ***p < 0.001 vs control. (**C** and **D**) Mitochondrial ROS production in H9c2 cells after culture in normal hypoxic conditions for 6 h with/without PRDX2 overexpression or down-regulation. The data are presented as means ± SD of the mean from ten independent views. ***p < 0.001 vs control. Magnification 200×. (**E**) The effects of PRDX2 on hypoxia-induced H9c2 cell viability were detected after exposed to hypoxia medium for 6 h. The data are presented as means ± SD of the mean from three separate cell experiments. *p < 0.05, **p < 0.01, ***p < 0.001 vs control. (**F**) Western blot analysis of Bcl-2, Bax, Caspase-3. GAPDH was used as a loading control. (**G**–**I**) Statistical analysis of band intensity for Bcl-2, Bax, and Caspase-3. The data are presented as means ± SD of the mean from three separate cell experiments. *p < 0.05, **p < 0.01, ***p < 0.001 vs control.

We further investigated the oxidative stress association of PRDX2 by silencing and overexpressing PRDX2 in H9c2 cells. Cells were transfected with PRDX2 siRNA (siPRDX2) and a scrambled control siSCR. The silencing effect on PRDX2 mRNA and protein levels were determined by RT-PCR and western blot after 36 and 48 h, respectively (Fig. 2A). Levels of mRNA and protein confirmed that PRDX2 had been reduced (*p* < 0.001 vs control). H9c2 cells were also transfected with a PRDX2 overexpression vector. PRDX2 mRNA and protein levels were assessed by RT-PCR and western blotting after 48 h and found to be significantly increased (*p* < 0.001 vs control) (Fig. 2B). Figure 2C shows mitochondrial ROS production in H9c2 cells measured by DCF fluorescence and cultured under normal or hypoxic conditions for 6 h with or without PRDX2 overexpression or downregulation. PRDX2 downregulation significantly increases the level of ROS production (*p* < 0.001) whereas ROS levels under hypoxic conditions remain unaltered when PRDX2 is overexpressed (Fig. 2D). PRDX2 overexpression also prevents loss of viability in H9c2 cells grown under hypoxic conditions (Fig. 2E). Western blot analysis of apoptotic-related proteins reveals that silencing PRDX2 leads to increased levels of Bax and Caspase-3 and reduced levels of Bcl-2, whereas, overexpressing PRDX2 counteracts these effects (Fig. 2F–I). These results demonstrate that PRDX2 expression inhibits hypoxia-mediated oxidative stress injury to H9c2 cells.

### PRDX2 expression induces TLR4-mediated VEGF and inflammatory factor expression under hypoxic conditions

**Figure 3:**
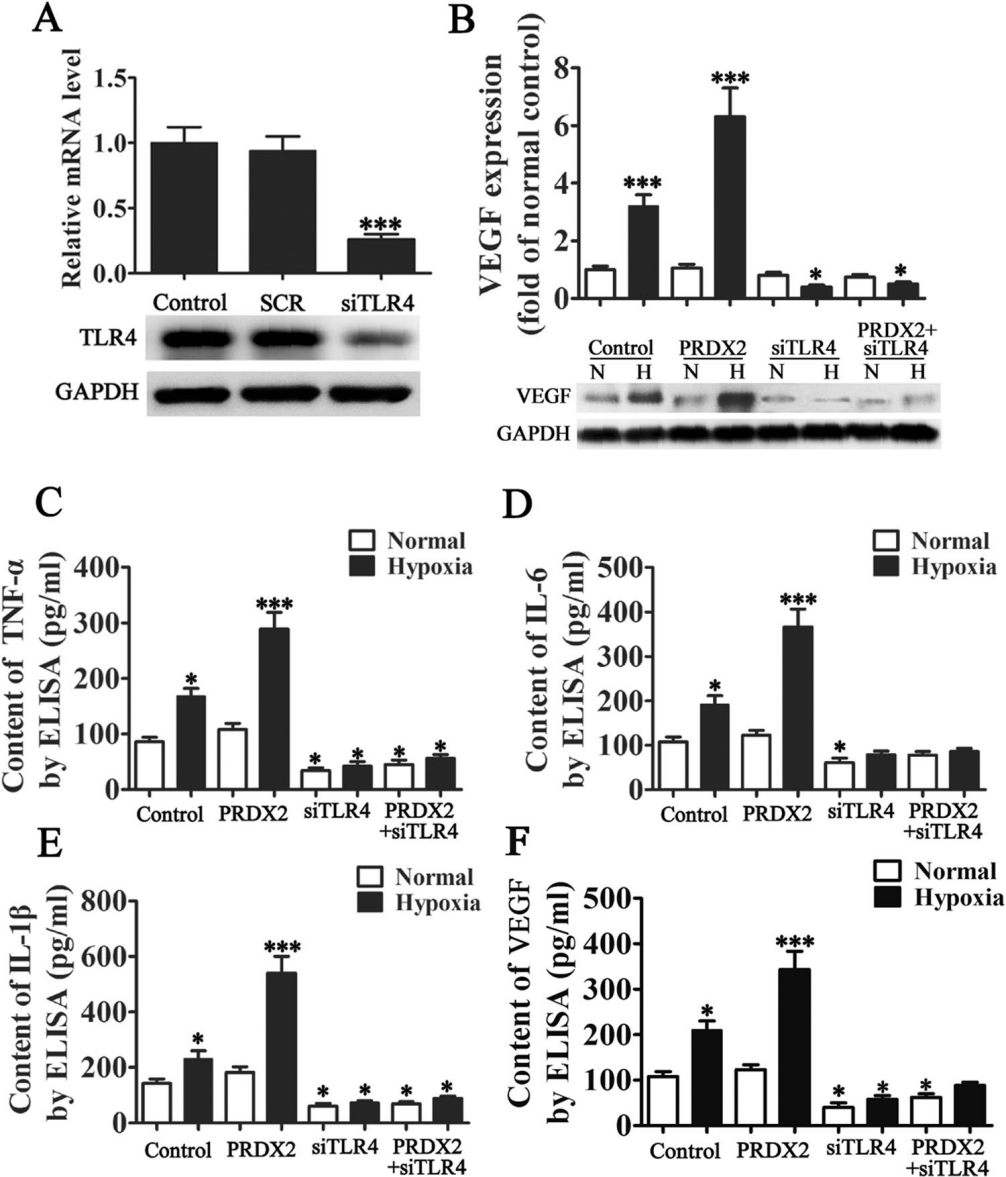
PRDX2 expression promote TLR4 mediated VEGF and inflammatory factor expression in hypoxia condition. (**A**) H9c2 cells were transfected with TLR4 siRNA (siTLR4) and control siRNA (siSCR) at a concentration of 80 pmols. Cells were harvested 36 h after the transfection, and the silencing effect on TLR4 mRNA levels was determined by RT-PCR. The silencing effect of TLR4 siRNA at the protein level was determined 48 h post-transfection using western blotting. GAPDH was used as a loading control. The data are presented as means ± SD of the mean from five separate cell experiments. ***p < 0.001 vs control. (**B**) Total protein was collected from H9c2 cells which were exposed in hypoxic condition for 6 h then cultured in normal condition for 24 h. The control group was exposed to normal conditions. VEGF protein levels were determined by performing western blot. The data are presented as means ± SD of the mean from three separate cell experiments. *p < 0.05, **p < 0.01, ***p < 0.001 vs control. (**C**–**F**) Supernatants from H9c2 cells were harvested and analyzed by enzyme-linked immunosorbent assay (ELISA) for TNF-α, IL-6, IL-1β and VEGF after exposure to hypoxic conditions for 6 h and then cultured in normal conditions for 24 h. Results are shown as pg/ml and are representative of three independent experiments. The data are presented as means ± SD. *p < 0.05, **p < 0.01, ***p < 0.001 vs control.

The impact of PRDX2 expression on TLR4-mediated VEGF activation was assessed in H9c2 cells. TLR4 expression was silenced in cells and expression levels of TLR4 and VEGF were determined by RT-PCR, western blot and ELISA (Fig. 3A,B,F). VEGF was induced in hypoxic conditions (*p* < 0.001) especially when PRDX2 was overexpressed (*p* < 0.001). However, levels of VEGF were reduced when TLR4 was silenced (*p* < 0.05) even when PRDX2 was overexpressed (*p* < 0.04). This confirms that the activity of VEGF was dependent on TLR4. Supernatants from H9c2 cells were harvested and analyzed by ELISA for levels of the cytokines TNF-α, IL-6 and IL-1β (Fig. 3C–E). Hypoxic conditions led to an increase in the level of all three cytokines (*p* < 0.05) and levels were increased further by the overexpression of PRDX2 (*p* < 0.001). Silencing TLR4 inhibited the expression of TNF-α, IL-6, and IL-1β even when PRDX2 was overexpressed. Taken together these results show that PRDX2 promotes the expression of VEGF, TNF-α, IL-6, and IL-1β but this interaction relies on the activity of TLR4.

### PRDX2 overexpression promotes HUVEC migration, vasculogenic mimicry formation and myocardial hypertrophy related protein expression

**Figure 4:**
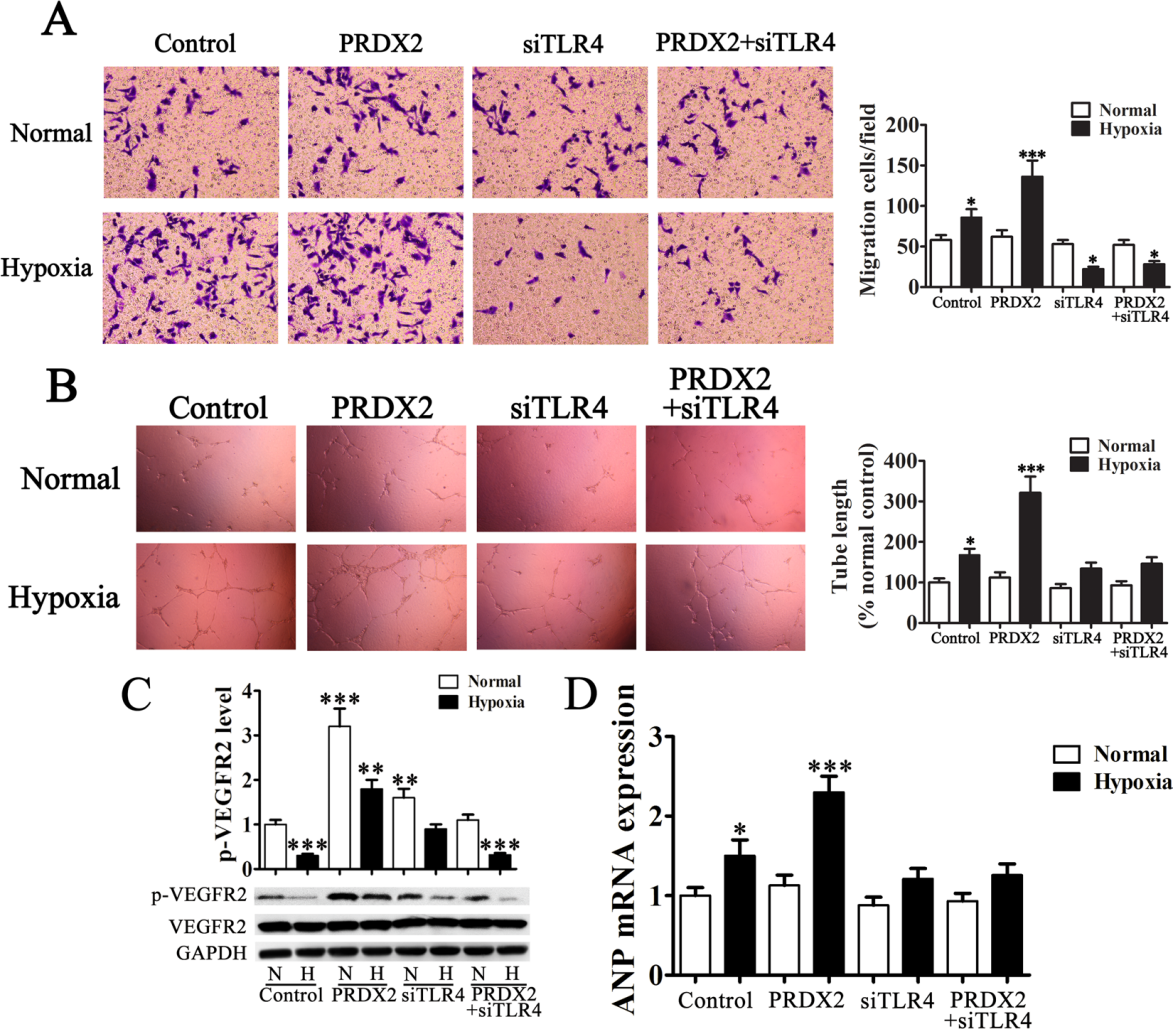
PRDX2 overexpression in H9c2 cells promote HUVEC migration, vasculogenic mimicry formation and myocardial hypertrophy related protein expression. H9c2 cells were cultured in serum-free medium and exposed to hypoxic conditions for 6 h then in normal conditions for another 24 h, then the medium was harvested for transwell and vasculogenic mimicry formation of HUVECs. (**A**) Migration of HUVECs was assessed using transwell chambers. The invasive capabilities of HUVECs are significantly increased with chemotaxis of PRDX2 overexpression in H9c2 cells. The data are presented as means ± SD of the mean from ten separate cell experiments. *p < 0.05, **p < 0.01, ***p < 0.001 vs control. (**B**) Images of tube formation. Tube length is presented as percent of total tube length per field versus untreated control cells. The data are presented as means ± SD of the mean from ten separate cell experiments. *p < 0.05, **p < 0.01, ***p < 0.001 vs control. (**C**) VEGFR2 phosphorylation in the surface of HUVECs was detected by western blots. The phospho-specific bands were quantified and normalized by the intensities of the corresponding VEGFR2 bands. The data are presented as means ± SD of the mean from three separate cell experiments. *p < 0.05, **p < 0.01, ***p < 0.001 vs control. (**D**) RT-PCR analysis of ANP expression. The data are presented as means ± SD from three separate cell experiments. *p < 0.05, **p < 0.01, ***p < 0.001 vs control.

H9c2 cells were cultured in serum-free medium and exposed to hypoxic and normal conditions, the media was then harvested for transwell and vasculogenic mimicry formation of HUVECs. Migration of HUVECs was assessed using transwell chambers (Fig. 4A). The invasive capabilities of HUVECs were significantly increased with PRDX2 overexpression in H9c2 cells (*p* < 0.001 vs control). However, migration was reduced when TLR4 was silenced in H9c2 cells even when PRDX2 was overexpressed. Similar results were obtained for tube formation with increased lengths in cells overexpressing PRDX2 under hypoxic conditions and no increase in length was found when TLR4 is silenced (Fig. 4B). PRDX2 increased migration and vasculogenic mimicry formation but this required the activity of TLR4.

We also assessed the phosphorylation of VEGFR2 on the surface of HUVECs by western blot (Fig. 4C). VEGFR2 phosphorylation was reduced under hypoxic conditions (*p* < 0.001) and similar levels were observed in cells with silenced TLR4 overexpressing PRDX2 (*p* < 0.001) under the same conditions. However, phosphorylation was increased in cells overexpressing PRDX2 under normal (*p* < 0.001) and hypoxic (*p* < 0.01) conditions. Phosphorylation was also increased in cells with silenced TLR4 under normal conditions (*p* < 0.01) and remained unaltered in hypoxic conditions compared with the control grown in normal conditions. RT-PCR analysis of atrial natriuretic peptide (ANP) expression was also determined (Fig. 4D). ANP mRNA levels were increased under hypoxic conditions (*p* < 0.05) and when PRDX2 was overexpressed (*p* < 0.001). No difference in ANP expression was detected when TLR4 is silenced under hypoxic conditions.

### Overexpression of PRDX2 inhibits ROS levels and myocardial injury after AMI but promotes inflammatory responses

**Figure 5:**
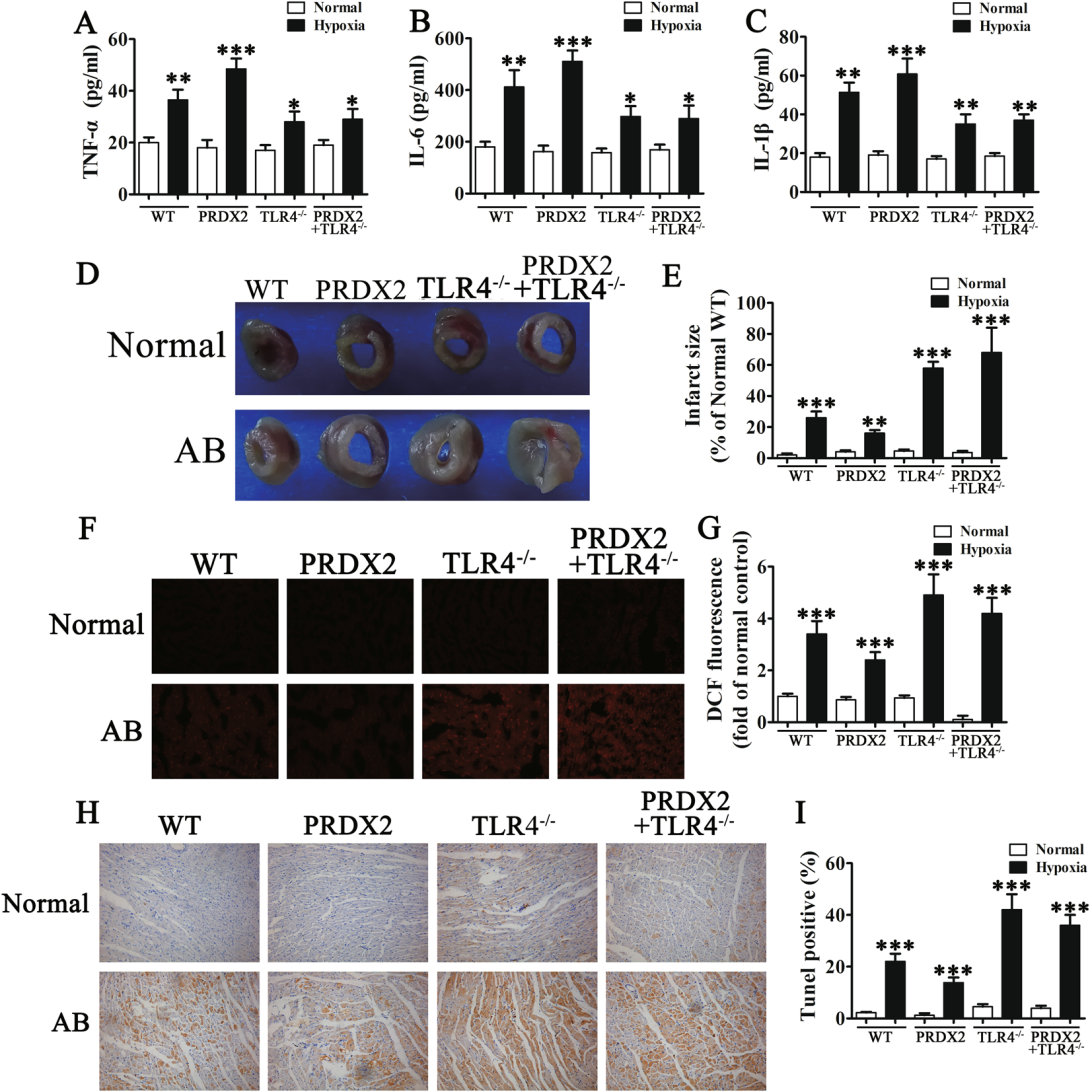
The overexpression of PRDX2 inhibits ROS levels and myocardial injury after AMI but promotes inflammatory responses. After 2 weeks of coronary artery ligation, serum from mice was harvested for ELISA analysis and after 1 month, myocardial tissue was used for immunohistochemical analysis. (**A**–**C**) ELISA for inflammatory cytokines. The data are presented as means ± SD from three separate experiments from five to six mice in each group. *p < 0.05, **p < 0.01, ***p < 0.001 vs control. (**D**) Representative images of infarct size as stained by TTC. (**E**) PRDX2 decreased infarct size compared with other groups. The data are presented as means ± SD from five to six separate mice in each group. **p < 0.01, ***p < 0.001 vs control. (**F**) Immunofluorescence for ROS generation by DCF-DA. (**G**) The results show that the expression of PRDX2 inhibits generation of oxidative stress and the inhibition of PRDX2 on ROS was enhanced with TLR4 knock out. The data are presented as means ± SD from ten separate fields. ***p < 0.001 vs control. (**H** and **I**) TUNEL analysis of apoptosis in myocardial infarction. Statistical analysis of apoptosis. AB, aortic banding. The data are presented as means ± SD from three separate experiments. ***p < 0.001 vs control.

We assessed the role of PRDX2 following myocardial injury in mice. After 2 weeks of coronary artery ligation, serum was harvested for ELISA and after 1 month, myocardial tissue was used for immunohistochemical analysis. Figure 5A–C shows the ELISA results for inflammatory cytokines TNF-α, IL-6, and IL-1β. All three cytokines were elevated under hypoxic conditions (All *p* < 0.01 vs control) particularly when PRDX2 was overexpressed (All *p* < 0.001 vs control), however, silencing TLR4 substantially reduced this increase (All *p* < 0.05 vs control).

Perhaps the most significant effect following myocardial injury in mice can be found in infarct size, shown in Figs. 5D,E. PRDX2 overexpression noticeably decreased infarct size compared with the other groups (*p* < 0.01). The highest infarct size was found in mice with TLR4 knockout (*p* < 0.001), in particular when PRDX2 was overexpressed (*p* < 0.001). We also assessed ROS generation in mouse tissue by DCF-DA and found that overexpression of PRDX2 inhibits the generation of oxidative stress although this effect was not observed in the TLR4 knock out (Fig. 5 F, G). We detected apoptotic cells in myocardial infarction tissue by using TUNEL analysis (Fig. 5H,I). The greatest cellular DNA damage and, therefore, highest percentage of TUNEL-positive cells was found in TLR4 knockout mice with the lowest percentage in mice with elevated levels of PRDX2 (All *p* < 0.001 vs control).

### PRDX2 overexpression promotes angiogenesis and myocardial hypertrophy

**Figure 6:**
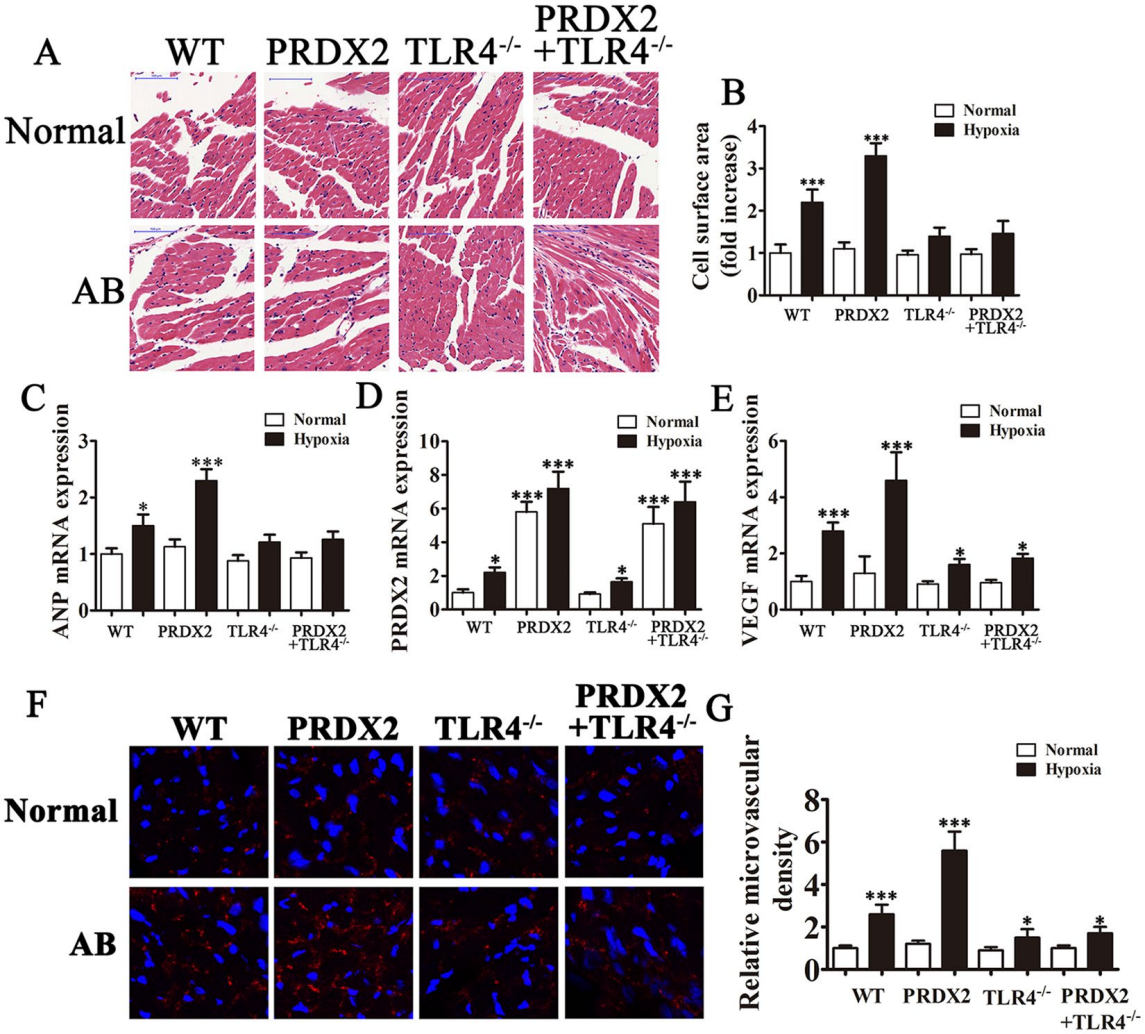
The overexpression of PRDX2 promote angiogenesis and myocardial hypertrophy. After 2 weeks of coronary artery ligation, serum from mice was harvested for RT-PCR analysis and myocardial tissue were harvested 4 weeks after artery ligation for immunohistochemical analysis. (**A**) Effects of PRDX2 on cell surface area of cardiomyocytes. (**B**) Cell surface area was calculated by measuring 10 random cardiomyocytes. (**C**–**E**) RT-PCR for ANP (**C**), PRDX2 (**D**) and VEGF (**E**) expression. The data are presented as means ± SD from three separate experiments from five to six mice in each group. *p < 0.05, ***p < 0.001 vs normal WT group. (**F**) The formation of microvascular cells in infarction region were stained with CD31. AB, aortic banding. The data are presented as means ± SD from 10 fields in each group. *p < 0.05, ***p < 0.001 vs normal WT group.

The effects of PRDX2 on the cell surface area of cardiomyocytes in mice following myocardial injury are shown in Fig. 6A. Cell surface area was calculated in 10 random cardiomyocytes and under hypoxic conditions was found to be greatest in mice overexpressing PRDX2 (*p* < 0.001) (Fig. 6B) suggesting that myocardial hypertrophy is promoted by PRDX2. Under normal conditions, there was no significant difference in cell surface area between control wild-type mice and those with TLR4 knockout, even when PRDX2 is overexpressed. Overall, this indicates that TLR4 is required for angiogenesis and myocardial hypertrophy.

Levels of ANP were highest in wild-type mice with hypoxia (*p* < 0.05) and mice overexpressing PRDX2 with hypoxia (*p* < 0.001). No difference in ANP levels was found in TLR4 knock out mice compared with normal mice (Fig. 6C). PRDX2 mRNA levels were highest in mice overexpressing PRDX2 (*p* < 0.001) and TLR4 knockout mice overexpressing PRDX2 (*p* < 0.001) (Fig. 6D) and VEGF levels were highest in mice with hypoxia e overexpressing PRDX2 (*p* < 0.001) and in wild-type mice with hypoxia (*p* < 0.001) (Fig. 6E). Similarly, the formation of microvessel in the infarction region was most dense in mice overexpressing PRDX2 with hypoxia and in wild-type mice with hypoxia. Overall, these results indicate that PRDX2 promotes angiogenesis and myocardial hypertrophy but this is mediated by TLR4.

## Discussion

Inflammatory responses repair the heart following AMI, however, elevated inflammation can lead to myocardial hypertrophy and heart failure^23, 24^. Patients with AMI can have a profound systemic inflammation response, involving inflammatory cytokines, chemokines, and cell adhesion molecules, and activation of peripheral leukocytes and platelets^25, 26^. Several studies have shown that this elevated inflammatory response can accentuate adverse remodeling in cardiac pathologic conditions, particularly in association with TLRs^27–29^. As with other studies, we found that serum concentrations of VEGF and the inflammatory factors IL-1β, TNF-α and IL-6 were increased in AMI patients, however, the expression of PRDX2 was also upregulated. Therefore, we concentrated our research on PRDX2 involvement in the inflammatory response under hypoxic conditions and following myocardial injury in addition to the more familiar role it has in protecting cells from oxidative stress. We also assessed PRDX2 participation in angiogenesis and myocardial hypertrophy and whether it may be mediated by TLR4.

We showed, through ELISA and *in vivo* experiments, that the expression of PRDX2 inhibits hypoxia-induced oxidative stress injury and reverses an apoptotic-related protein profile involving caspase-3, Bax, and Bcl-2. PRDX2 down-regulation significantly increased the level of ROS production and reduces cell viability, whereas, the opposite occurs when PRDX2 is overexpressed. Furthermore, we demonstrate that overexpression of PRDX2, in turn, promotes the expression of VEGF, TNF-α, IL-6, and IL-1β but this influence relies on the activity of TLR4. It is thought that PRDX2 acts as a redox-dependent inflammatory mediator that mediates the release of TNF-α through macrophages^30^. The PRDX2 substrate thioredoxin is also released by macrophages and together they are thought to modify the redox status of cell surface receptors to facilitate their activation via cytokine and TLRs and thereby induce an inflammatory response^30^.

PRDX2 overexpression in H9c2 cells also increases HUVEC migration, vasculogenic mimicry formation and myocardial hypertrophy related protein expression. Whereas, the overexpression of PRDX2 inhibits ROS level and alleviates myocardial injury after AMI but promotes inflammatory responses *in vivo*. In addition, immunocytochemistry and immunofluorescence analysis showed that overexpression of PRDX2 promotes angiogenesis and myocardial hypertrophy. Taken together, our results indicate that PRDX2 plays two roles: it increases cell survival in hypoxia on the one hand but contributes to inflammatory myocardial hypertrophy in acute infarcted on the other. Similar results have been reported in the literature, extensive experimental evidence demonstrates that induction of the inflammatory response can have dual function of myocardial injury and repair^31–34^. Furthermore, antibody neutralization of adhesion molecules, chemokines and cytokines reduces the size of the infarct following myocardial injury by 40–50%^35^. Impaired or silenced TLR4 signaling inhibits the inflammatory reaction following myocardial infarction and reduces adverse remodeling^28, 36^. In this work, the overexpression of PRDX2 was able to reduce infarct size, however, the silencing of TLR4 increased infarct size. We also found that overexpression of PRDX2 increased migration and vasculogenic mimicry formation but this was facilitated by TLR4. PRDX2 is associated with tumor progression and has been implicated in the metastasis of cancers through its interactions with TGFβ1-induced epithelial-mesenchymal transition^37^. Alternatively, PRDX2 is thought to assist cancer progression through protecting metastatic cells from oxidative stress^38^. Further investigation could enable the mechanism by which PRDX2 is involved in cell migration to be understood more clearly.

In this study, we demonstrate that, as with PRDX1 and PRDX6, PRDX2 has an ischemic or hypoxic protective role. In mouse vascular endothelial cells, PRDX1 causes increases in VEGF expression that is dependent upon TLR4^39^. PRDX1-mediated activation of the TLR4/NF-κB pathway and the production of TNF-α, IL-6 and IL-17 are stimulated by neuroinflammatory injury in intracerebral hemorrhage^40^. Furthermore, hypoxia-induced adverse reactions can be reversed in retinal ganglion cells by the over-expression of PRDX6^41^. Whether, PRDX1 and PRDX6 are involved in myocyte hypertrophy in AMI would be an interesting area for further research.

To conclude we find that PRDX2 involvement in myocyte hypertrophy and survival is mediated by TLR4 in AMI. Overexpression of PRDX2 inhibits ROS level and myocardial injury after AMI but promotes inflammatory responses *in vivo*. By immunocytochemistry and immunofluorescence, we have shown that overexpression of PRDX2 promotes angiogenesis and myocardial hypertrophy. Taken together, our results indicate that PRDX2 promotes cell survival but is also involved in inflammatory myocardial hypertrophy in acute infarcted.
